# Bioanalytical LC-QTOF/MS Method for a *N*-phenylpiperazine Derivate (LQFM05): An Anxiolytic- and Antidepressant-like Prototype Drug Applied to Pharmacokinetic and Biodistribution Studies

**DOI:** 10.3390/ph16070930

**Published:** 2023-06-26

**Authors:** Ana Cláudia M. Ramos, Kênnia R. Rezende, Carolina M. Teixeira, Aline R. Fernandes, Heloa Santos, Rúbia Darc Machado, Ricardo Menegatti, Boniek G. Vaz, Andréa R. Chaves

**Affiliations:** 1 Laboratory of Biopharmacy and Pharmacokinetics (BioPK), Faculty of Pharmacy, Federal University of Goiás, Goiânia 74605-170, GO, Brazil; 2 Laboratory of Chromatography and Mass Spectrometry (LaCEM), Chemistry Institute, Federal University of Goiás, Goiânia 74690-900, GO, Brazilboniek@ufg.br (B.G.V.); 3 Laboratory of Pharmaceutical Medicinal Chemistry (LQFM), Faculty of Pharmacy, Federal University of Goiás, Goiânia 74605-170, GO, Brazil

**Keywords:** antipsychotic drug LQFM05, sample pretreatment, tissue distribution, bioanalytical methods, LC-QTOF/MS

## Abstract

The LQFM05 is a prototype drug designed for treatment of psychiatric disorders, such as schizophrenia, exhibiting anxiolytic- and antidepressant-like (12 or 24 µmol/kg) effects in classical behavioral tests. In order to evaluate its pharmacokinetic properties, a liquid chromatography method coupled to a quadrupole time of flight mass spectrometry system (LC-QTOF/MS) was developed and fully validated for LQFM05 analysis in rat plasma and tissue samples (brain, heart, liver, and kidneys). Liquid–liquid extraction, solid phase extraction and protein precipitation were assessed as clean-up procedures for biological samples and analyte enrichment. Plasma and tissue samples underwent protein precipitation as a preliminary step, using acetonitrile. Linearity was fully demonstrated for the dynamic range (10.0 to 900.0 ng/mL), with r^2^ values higher than 0.99 (RSD_slope_ ≤ 2%, F_cal_ < F_tab_, C_cal_ < C_tab_). Biodistribution studies in rats revealed high brain tissue concentrations (12.4 µg/g), suggesting elevated drug affinity to the main therapeutic target tissue, showing a blood partition coefficient of 1.9. Kidneys also showed great exposure and tissue affinity, suggesting a potential extrahepatic clearance. Likewise, all examined tissues exhibited satisfactory LQFMF05 distribution. The mass fragmentation spectrum indicated the presence of its main metabolite, LQFM235, yielded by high hepatic hydroxylation route, an equally bioactive derivative. Lastly, the developed LC-QTOF/MS method was shown to be sensitive (LOQ = 10 ng/mL), precise and accurate for LQFM05 determination in tissue homogenates and plasma samples.

## 1. Introduction

Schizophrenia is a severe chronic psychiatric disorder and one of 25 leading causes of disability worldwide [1,2]. Clozapine is the most used drug for schizophrenia disorder control, and is therefore constantly related to hematological and metabolic disorders as a side effect for some clinical patients [3], limiting its prescription and use. To fill this gap, 1-(4-methoxyphenyl)−4-((1-phenyl-1H-pyrazol-4-yl)methyl)piperazine (LQFM05) was investigated as a promising prototype antipsychotic *N*-phenylpiperazine drug, showing anxiolytic- and antidepressant-like effects in classical behavioral tests (12 or 24 µmol/kg LQFM005), putatively through activation of 5-HT1A receptors [4].

Preclinical pharmacokinetics studies are key aspects of the new drug development phase as a way of identifying an adequate bioavailability profile, tissue distribution and excretion rates. Furthermore, it can support data on dosage schedules, pharmacological information, and possible toxicity [5,6,7]. In this sense, a reliable and accurate analytical method is required.

Several methods for quantification of phenylpiperazine antipsychotics and atypical antidepressants have been reported, often using liquid chromatography (LC) with ultraviolet (UV), diode array (DAD), fluorescence (FLD) and mass spectrometry detector (MS) [8,9,10,11]. 

Moreover, a sample pretreatment procedure also seems like a crucial step for analytical method development. Techniques such as protein precipitation, liquid–liquid extraction (LLE), solid phase extraction (SPE), solid phase microextraction (SPME) and hollow fiber-based liquid phase microextraction (HF-LPME) have been used to extract and/or pre-concentrate drugs in the biological fluid before analytical system analysis [9,12,13,14,15].

Mass spectrometry methods have several applications in pharmacokinetic studies due to the better selectivity of the higher resolution mass spectrometers, such as the quadrupole time-of-flight mass spectrometry system (QTOF/MS). The higher resolution, sensitivity and selectivity of these systems, especially when coupled to a liquid chromatography system (LC-QTOF/MS), allow for techniques with reduced time consumption and sample pretreatment costs.

Thus, here, we present a bioanalytical LC-QTOF/MS method applied to the pharmacokinetics and biodistribution studies of a new potential antipsychotic drug with anxiolytic and antidepressant-like effect (LQFM05) using a straightforward sample pretreatment procedure. Pharmacokinetics and drug tissue/plasma ratio (Kp) to the brain, heart, liver and kidneys were also evaluated. 

## 2. Results and Discussion

### 2.1. Method Development and Sample Preparation Strategies

**Figure 1:**
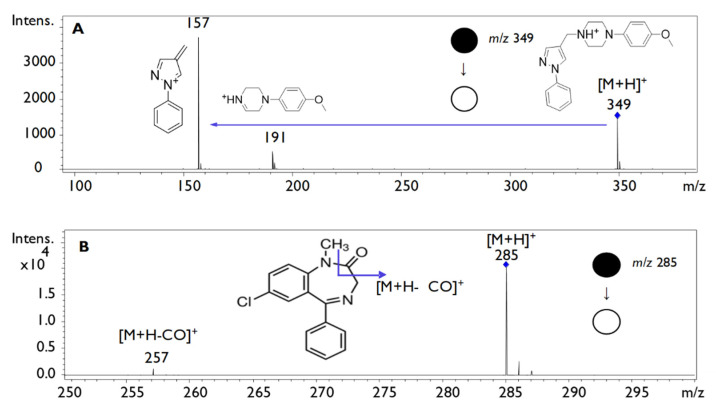
MS/MS spectrum of (**A**): LQFM05 at 30 ng/mL (retention time at 6.3 min), [M + H]^+^ 349 *m*/*z*, and (**B**): diazepam 250 ng/mL (retention time at 5.6 min), [M + H]^+^ 285 *m*/*z*.

Carbamazepine, dexamethasone, olanzapine, ethinylestradiol, fluoxetine macohydrochloride and diazepam were evaluated as internal standards (ISs) because some of their physicochemical properties, such as log P and parent fragmentation in the MS/MS spectrometer, were similar to LQFM05 [16,17]. Accordingly, diazepam was selected for MS ionization condition, fragmentation profile, matrix endogenous compounds interference, as well as chromatographic profile. The LQFM05 and diazepam MS/MS obtained spectrum are shown in Figure 1. The most abundant ion (157.08 *m*/*z*) was selected as the LQFQ05 quantifier.

**Figure 2:**
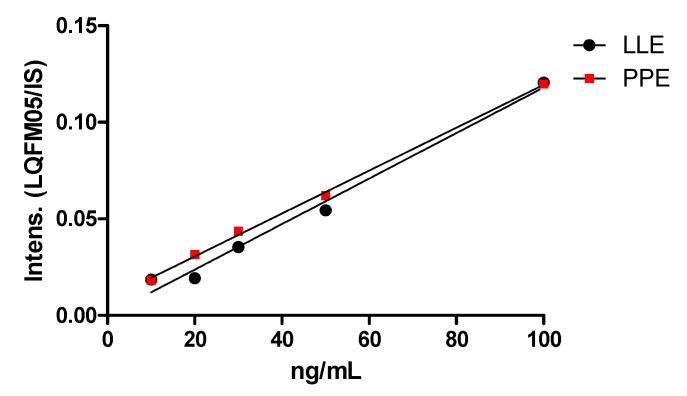
LLE and PPE calibration curves (10.0–100.0 ng/mL, *n* = 5) in liver homogenate.

For sample clean-up and analyte enrichment procedures, solid phase extraction (SPE), liquid–liquid (LLE) and protein precipitation extraction (PPE) were evaluated. Some SPE drawbacks may include multiple steps and slow filtration. Moreover, the observed analyte run time elution was inadequate (~20 min) as well as its selectivity. Instead, LLE was shown to be a faster and less costly sample preparation procedure than SPE. Experiments were performed based on the protocol established by Kumar and Ramanathan [10], although considerable residual effects were observed. Eventually, PPE was also evaluated. Regardless of some often-reported disadvantages, such as low selectivity and ion signal suppression, herein data from PPE procedure showed better sample clean-up, with no MS signal interferences. In summary, the efficiency of the evaluated techniques is expressed by a slightly higher determination coefficient (r^2^) for PPE (0.9985) compared to LLE (r^2^ = 0.9872), as exemplified by the liver extraction procedure in Figure 2.

### 2.2. Analytical Validation

**Figure 3:**
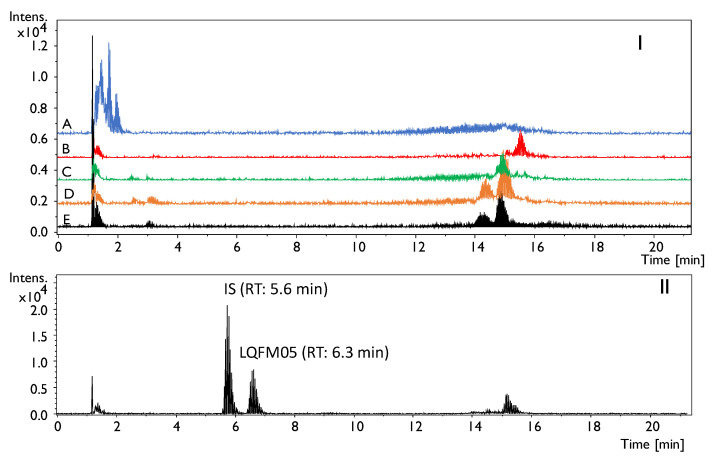
Total ion chromatogram (TIC) of: (**I**) reference blank samples (A: plasma; B: brain; C: liver; D: kidneys and E: heart); (**II**) heart homogenates (IS: 250 ng/mL and LQFM05: 250 ng/mL).

The total ion chromatograms (TICs) of blank plasma, brain, heart, liver and kidney blank samples and heart compared to matrix spiked with IS (250 ng/mL) and LQFM05 (10 ng/mL) are shown in Figure 3. LQFM05 and IS retention time (RT) were 5.6 min and 6.3 min, respectively. Endogenous or exogenous interferences were not seen for LQFM05 and IS peaks in the matrices studied.

Analytical parameters assessed during the LC-MS method validation process (linearity, precision, accuracy, etc.) aim to provide evidence of confidence for the applied sample preparation and analysis procedure. Methods of calibration are especially meaningful for assaying studies. Linearity in the LQFM05 concentration range of 10.0 to 900.0 ng/mL was primarily assessed by means of an r^2^ > 0.99. The lower limit of quantification (LLOQ) concentration was at 10.0 ng/mL based on the signal/noise higher than 10 with accuracy and precision. A summary of these values can be found in Table 1.

In general, the relative standard deviation of the slope (RSDslope) used to evaluate linearity and the significance of the angular coefficient is an indication of the experimental dispersion data around the regression line [20]. The RSDslope values in all matrices are in the range of 0.47 to 1.6% (Table 1). According to the obtained RSDslope values, the regressions can be considered linear (≤2%) [20]. 

Additionally, the Cochran hypothesis test was performed in order to evaluate the homogeneity of variance of the residues from the (y) axis. In other words, if the calculated (Ccal) value at a 5% level of significance (P = 0.05) found for (J = 4 and I = 10) was lower than 0.373 (Ctabulated). As the calculated value (Ccal) was lower than Ctabulated in all the matrices, there is homoscedasticity of responses [19,20]. Hence, once the hypothesis was accepted, the least squares method was adequate for estimating the best regression line that passes through the points obtained experimentally from the calibration curve.

Fisher-Snedecor’s F-Ratio was calculated for comparison between calibration data variability. At the 95% confidence level, F-tabulated (0.05, 8, 30) for 10 concentrations levels (I) and two different days were 2.27 [18,19,20]. Accordingly, Fcal was lower than Ftab in all matrices, and the obtained results describe a linear calibration. See Table 1.

**Figure A1:**
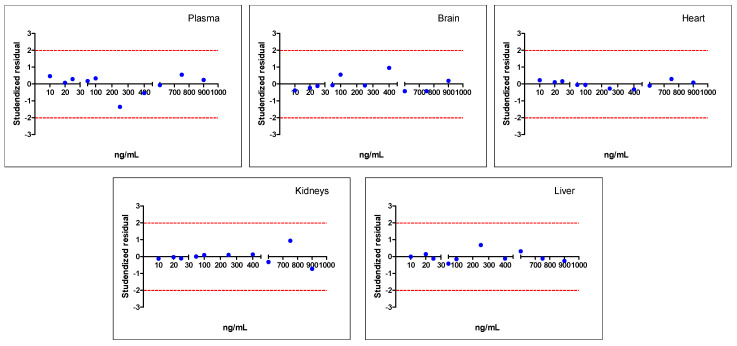
Studentized residues of LQFM05 in rat plasma and tissue homogenates (brain, heart, liver and kidneys) obtained from the calibration curve by LC-QTOF/MS.

Graphical representation of the residues between observed and predicted Y (response) values is also a very important procedure for detection of outliers, points of influence, lack of adjustment or unequal variation. However, Jurado et al. [21] adds the differentiation of the use of studentized residuals by the residual standard deviation (S_y/x_) and the leverage effect of each point on the regression line. Studentized residues (Figure A1) distributed around zero showed no tendency and corresponded to the acceptance criteria (±2) in the range of analyzed concentrations. Thus, the regression model used was considered adequate to this aim.

The intra- and inter-assay precision and accuracy were estimated at four concentration levels, each concentration in replicates (*n* = 6 and *n* = 12, respectively). All results are shown in Table 2. 

Recovery of a bioanalytical method measures efficiency of the extraction procedure. Regarding drug recovery rates from all investigated tissues, the lowest one was found for the brain (around 45%), probably as a result of the high drug affinity to brain tissue. Additionally, biotransformation by enzymatic route can also bias to lower recovery rates. However, is worth mentioning that high analytical precision was found at all drug concentration levels (RSD% ≤ 10.9%), providing reliable recovery values. See Table 3.

The IS matrix effects showed values in the range of 0.98 (30 ng/mL: heart and liver) to 1.09 (30 ng/mL: plasma, and RSD% ≤ 8.4% (Table 3) in agreement to worldwide acceptance criteria [22]. Thus, results imply no significant drug ionization suppression or enhancement in plasma or other homogenates [23]. All RSD% values were lower than 15%.

Adsorption of the analyte or IS into the injector, chromatographic column, LC connections, ionization interface or other section of the chromatographic system can result in a late peak appearance of them into the next following run [24]. Potential interfering peaks at the analyte retention time should not exceed 20% of the obtained value when setting the LLOQ. In the present developed method, no carryover was observed at retention time of both IS (5.6 min) and LQFM05 (6.3 min) after injecting the highest calibrator concentration (900.0 ng/mL). 

**Figure 4:**
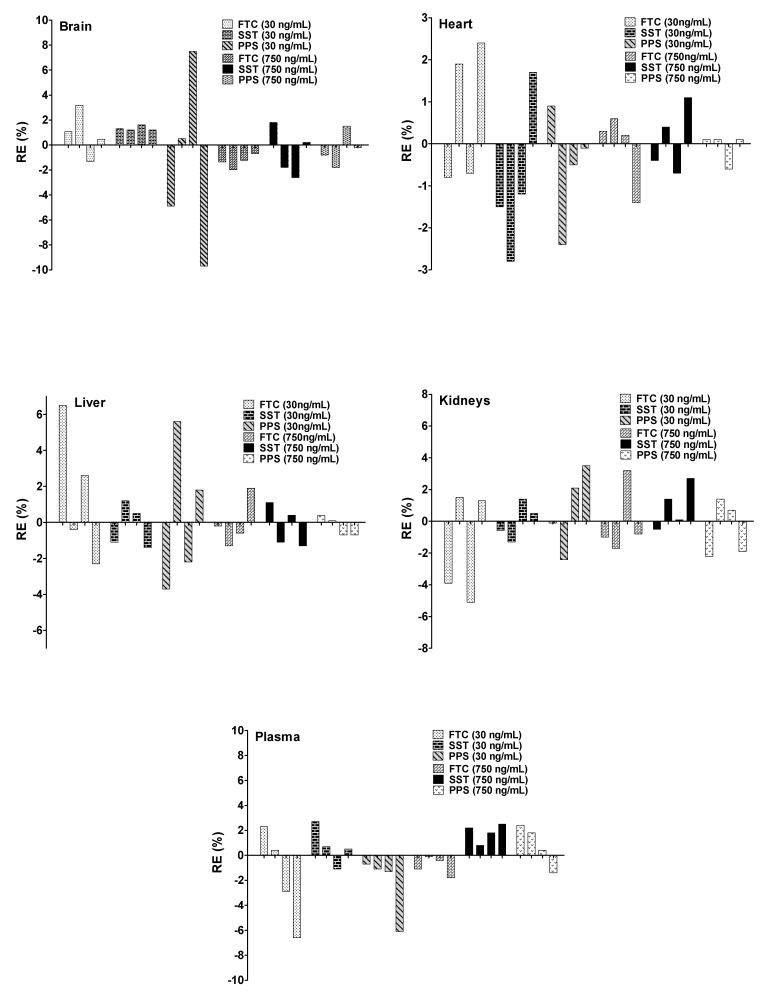
LQFM05 and IS stability in rat plasma and tissue homogenates (brain, heart, liver, kidneys) under different storage conditions (*n* = 4) by LC-QTOF/MS compared to fresh samples. FTC: stability under freezing and thaw cycles; SST: stability after short time analysis; PPS: postprocessing analysis; RE%: relative error.

The stability was assessed at three different conditions (FTC: stability after freezing and thawing cycles, SST: short time stability and PPS: post-processing stability). The obtained data showed acceptable accuracy and precision values below 15% (Figure 4) [22]. 

### 2.3. Tissue Distribution Study

**Figure 5:**
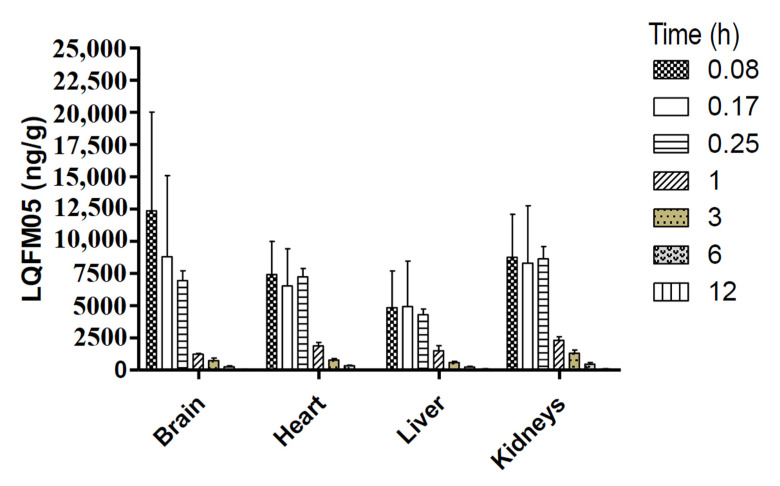
LQFM05 concentration-time profile in four tissue homogenates after an *i.v.* administration (10 mg/Kg) to rats. The final amount (ng/g) was calculated after considering the appropriate dilution factor.

Tissue distribution of LQFM05 was investigated in male Wistar rats at 0.08, 0.17, 0.25, 1, 3, 6 and 12 h after *intravenous* (*i.v*). dosing (10 mg/Kg) by collecting tissue samples from the brain, heart, liver, kidneys and blood. The concentration-time profile of LQFM05 in various tissues is shown in Figure 5. 

Non-compartmental analysis (NCA) was employed without assumption of any previous PK model, as it could be used in all cases, being the main advantage of the method [25]. All tissues investigated (Table 4) showed decreasing tissue concentrations from 0.08 to 12 h, except for liver tissue (t_max_ at 0.17 h). The highest tissue concentration (Figure 5) was obtained in the brain tissue (12,357.0 ng/g), indicating higher tissue affinity as desired to any psychoactive drug candidates.

The highest exposure to LQFM05 was found in the kidneys (14,595.2 h*ng/g) followed by the heart (10,919.6 h*ng/g) and brain (10,460.6 h*ng/g). The lowest exposure was observed in the liver (8235.3 h*ng/g) in a similar manner as that found for the LASSBio-579 [26].

The liver and brain half-lives were higher (3.6 and 2.5 h, respectively) compared to other tissue values around to 2.2 h. The largest mean residence time (3.1 h) was observed in the liver. Penetration coefficient values (Kp) were larger for the kidneys (2.7), heart (2.0) and brain (1.9). Thus, according to Kp data, LQFM05 was widely distributed to tissues (Kp > 1). Similar results were found for phenylpiperazine analogs, such as LASSBio-579 and 581, except for adipose tissue, which presented a value of 2.24 for penetration of LASSBio-579 [8,11]. 

Although LQFM05 penetration was much larger when compared to LASSBio-579 (log P 3.48) and LASSBio-581 (log P 2.91), it can be related to its higher lipophilicity (log P 3.68), favoring drug passage through biological phospholipid bilayer membranes. Other works also reported Kp > 1 as a tissue penetration pharmacokinetic parameter. In order to compare different formulations of quetiapine, an atypical antipsychotic, Carreno et al. [27] reported values ranging from 1.5 for the brain to 3.6 for the liver, according to the formulation administrated; Laxman et al. [28] also explores the tissue affinity of a new inhibitor, with Kp values ranging from 0.03 in the brain to 36.33 in the small intestine, where it showed remarkable accumulation. 

**Figure 6:**
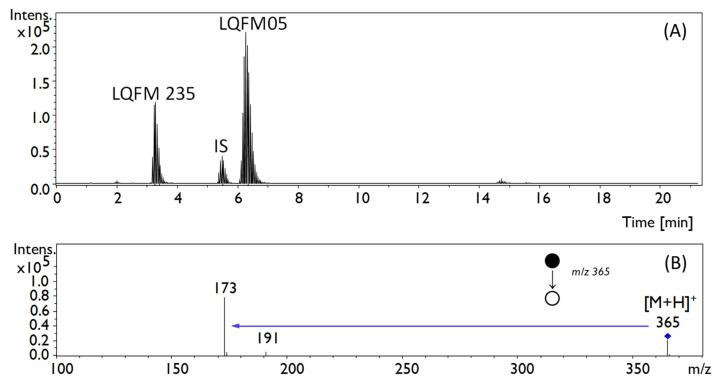
(**A**) Total ion chromatogram (TIC) of liver homogenate sample at 0.17 h; (**B**) fragmentation spectrum of LQFM235 (retention time: 3.3 min) by LC-QTOF/MS. MS spectra characterization in [4].

In this sense, supplementary LC analysis of rat tissue samples also detected the 4-(4-((4-(4-methoxyphenyl)piperazin-1-yl)methyl)−1H-pyrazol-1-yl)phenol (LQFM235) [4], a bioactive metabolite of the LQFM05 at 3.3 min (Figure 6). 

As a metabolite, it is assumed to be a less hydrophobic compound than LQFM05 and more easily eliminated from the body. Although the metabolite quantification was not the main purpose of the present study, the LQFM235 concentration was tentatively estimated in all tissues from *in vivo* rat samples (Figure 6) as it also showed anxiolytic-like properties, as previously demonstrated [4]. Both LC-MS drug analyses were run under the same experimental conditions, meaning that the same sample preparation and instrumental conditions were used (Section 3.4.2), except for the MRM transition ions of LQFM235 (Figure 6). The putative average area of the chromatographic peak was a direct comparison to a LQFM235 (500 ng/mL, *n* = 3) peak area obtained from an original sample (not fully validated method).

**Figure 7:**
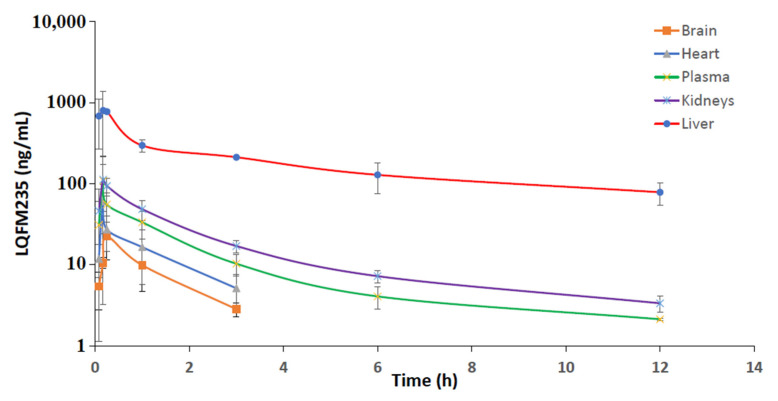
Putative mean tissue concentration (±tandard deviation)—time curves of metabolite LQFM235 after *intravenous* administration of LQFM05 (10 mg/kg).

From the biodistribution study, the metabolite could be detected and tentatively quantified in all tissues (Figure 7), with the exception of brain and heart matrices after 6 and 12 h post LQFM05 administration. The liver had the highest LQFM235 concentration at all sample times as compared to other evaluated tissues.

## 3. Materials and Methods

### 3.1. Chemicals and Materials

Diazepam (IS), purity ≥99%, was purchased from the National Institute for Quality Control in Health (Rio de Janeiro, Brazil). Acetonitrile (ACN) and methanol (MeOH) of LC-grade were obtained from Merck (Darmstadt, Germany). Ammonium acetate and hydroxypropyl-β-cyclodextrin were purchased from Sigma-Aldrich (St. Louis, MO, USA). Ultrapure water (≥18 M Ω cm) was obtained from an Elga Purelab Option-Q water purification system (Elga, Purelab Option-Q, EUA). 

LQFM05 was synthesized at Laboratório de Química Farmacêutica Medicinal (LQFM, Universidade Federal de Goiás, Goiânia, Brazil). Purity (>98% ± 0.95 *w*/*w*; *n* = 2) was determined by ^1^H spectra, as described elsewhere [4].

### 3.2. Animals

Male Wistar rats weighing 300 ± 50 g (10–12 weeks of age) were provided from the University Animal House (UFG, Goiânia, Brazil). The experimental protocol (022/17; 22 August 2017) was approved by the Ethics Committee on the Use of Animals of Universidade Federal de Goiás (Goiânia, Brazil). One week before experiments, animals were acclimatized under controlled conditions of temperature (24 ± 2 °C), humidity (50 ± 10%) and 12 h light-dark cycle, with free access to water and standard laboratory rodent food.

### 3.3. LQFM05 Dosage Form Preparation

LQFM05 (*ca.* 600.0 mg) was dissolved in 50 mL of 30% (*w*/*w*) hydroxypropyl-β-cyclodextrin (HP-β-CD) aqueous solution and shaken in a borosilicate flask (1500 rpm, 24 h at 21 ± 1 °C). The sample was filtered and sterilized (Nylon filter; 0.22 µm) to obtain a clear LQFM05 drug solution of 11.87 ± 0.44 mg/mL (*n* = 6) prior to *in vivo* analysis. The concentration of LQFM05 was determined by the validated LC-QTOF/MS method over the linear concentration range (0.0001–0.08 mg/mL). 

#### 3.3.1. Drug Administration to Pharmacokinetic Studies 

A single dose of LQFQM05 solution was administered intravenously (IV, 10 mg/kg) and orally in two different doses (20 and 40 mg/kg, max vol. 0.4 mL/100 g body weight) to groups of four healthy male Wistar rats: G1 (10 mg/mL, *n* = 4) received an intravenous bolus dose (ca. 30 sec bolus injection; ca. 0.1 mL/100 mg BW) via catheterization of the carotid artery and, G2 (20 mg/Kg, *n* = 4; *ca.* 0.2 mL/100 mg BW) and G3 (40 mg/kg, *n* = 4; ca. 0.4 mL/100 g BW) received a single oral dose by gavage. 

Blood samples (150–200 µL) were withdrawn from the lateral tail vein (max 6% BW) at 0.08; 0.25; 0.50; 0.75; 1.0; 1.5; 2.0; 2.5; 3.0; 4.0; 5.0 and 6.0 h after IV drug administration. After oral dosing, samples were taken at 0.17; 0.33; 0.50; 0.75; 1.0; 1.50; 2.0; 3.0; 4.0; 6.0; 9.0 and 12.0 h. The study design was based on a LQFM05 pilot study and previous work from Tasso et al. (2005) [11] and Conrado et al. (2008) [26]. 

Pharmacokinetic parameters were estimated by using non-compartmental analysis (NCA) without the assumption of any previous PK model and performed using Phoenix (WinNonlin^®^ version 8.1; Pharsight Corp., Mountain View, CA, USA), plus Microsoft Excel software.

#### 3.3.2. Drug Administration to Tissue Distribution Study

For the LQFM05 tissue distribution study, 21 rats were randomly divided into seven groups. After *i.v*. dosing (10 mg/kg; 28.7 µmol/kg), biological fluids and tissue samples (heart, liver, kidneys, brain and blood) were collected from animals at 0.08, 0.17, 0.25, 1, 3, 6 and 12 h post-dosing time intervals. 

The LQFM05 concentration in rat tissues was expressed in ng/g and calculated by Equation (1) [11,26].

C_t_ = (C_s_ × Vs)/P (1)

where C_t_ represented the tissue concentration (ng/g), and C_s_, V_s_ and P were the concentration (ng/mL), volume (mL) and weight (g) of the tissue samples, respectively.

### 3.4. Preparation of Samples for LC-QTOF/MS Analysis

#### 3.4.1. Stock Solutions, Calibration Standards and Quality Control Samples 

Aiming to provide evidence of confidence for the applied sample preparation and analysis procedure, LQFM05 and diazepam (IS) stock solutions were prepared in acetonitrile (ACN 1.0 mg/mL). Next, the stock was diluted to yield working solutions in ACN-water (1:1 *v*/*v*). Calibration curve samples (10.0, 20.0, 25.0, 50.0, 100.0, 250.0, 400.0, 600.0, 750.0 and 900.0 ng/mL) were prepared by spiking blank plasma/tissue samples with equal volumes of standard working solutions aliquots. Quality control (QC) samples (10.0, 30.0, 450.0, 750.0 and 1500.0 ng/mL) were prepared separately from a different drug weighting batch. The IS working solution (250 ng/mL) was prepared from stock standard solution in ACN. 

For LC-QTOF/MS analysis, the sample residue was reconstituted with ACN:10 mM ammonium acetate (1:1, *v*/*v*, 100 μL), vortexed (10 min), centrifuged (12,000 rpm/12 min) and then injected into an LC system (15 μL).

#### 3.4.2. Unkown Rat Plasma and Tissue Samples

Blood was collected in heparinized tubes and immediately centrifuged (1000 rpm/111× *g*, 10 min) to separate the plasma portion. The supernatant was transferred into clean tubes and stored at −80 °C until analysis by LC-QTOF/MS. Tissue samples were gently blotted with absorbent paper to remove the excess blood, weighed, and stored at −80 °C until analysis. 

Plasma, calibration standards and QC samples were processed by protein precipitation. Previously, plasma (100 μL) was spiked with IS (100 μL, 250 ng/mL) and deproteinized with ACN (500 μL), vortexed (10 min), centrifuged (10,000 rpm/10 min) and the supernatant was transferred to clean 1.5 mL tubes and vacuum dried (45 °C, 90 min). Tissue samples from biodistribution studies were first homogenized (Ultra-Turrax^®^ model T10 basic, IKA, Staufen, Germany) in 10 mM monobasic potassium phosphate buffer (pH 9.0) at the ratio of 1:3 (organ: buffer, *w*/*v*), except for liver which was 1:2 (*w*/*v*). After, IS solution (100 μL, 250 ng/mL) was added to tissue homogenates (100 μL) and submitted to extraction as previously mentioned. If any of the quantified values were higher than ULOQ, an appropriate sample aliquot of supernatant was additionally diluted using the same biological matrix as an actual sample. The final amount (ng/g) was calculated after considering the dilution factor.

For LC-QTOF/MS analysis, the sample residue was reconstituted with ACN:10 mM ammonium acetate (1:1, *v*/*v*, 100 μL), vortexed (10 min), centrifuged (12,000 rpm/12 min) and then injected into an LC system (15 μL).

### 3.5. Instrumentation

#### LC-QTOF/MS Conditions

Data were acquired on a Shimadzu LC liquid chromatography system (LC-20AD, Shimadzu Corporation, Kyoto, Japan) with two pumps (LC-20AD), an autosampler (SIL-20ACHT) and a UV detector (SPD-20AV) and also coupled to a micrOTOF-Q III mass spectrometer (Brucker Daltonics, Bremen, Germany) equipped with electrospray source (ESI) operated in a positive-ion mode (ESI(+)) using Brucker Data Analysis software. Data acquisition was carried out in the “multiple reaction monitoring” (MRM) mode, and the most abundant ion was selected as the quantifier ion. Recall that TOF detectors simultaneously collect both precursor and product ion information over a mass range without isolating a particular ion. The two most intense product ions of LQFM05 were selected as quantifier (349.21 *m*/*z* → 157.08 *m*/*z*) and qualifier ions (349.21 *m*/*z* → 191 *m*/*z*). MS parameters of the analyte and IS are listed in Table 5.

Chromatographic separation was performed using the X-Terra RP18 column (150 × 3.0 mm, 3.5 μm, Waters), at 40 °C. The binary mobile phase of 10 mM of ammonium acetate (A) and ACN (B) with gradient elution: 0–8 min (55% A), 8–9 min (50% A), 9–10 min (45% A), 10–11 min (40% A), 11–12 min (35% A), 12–13 min (30% A), 13–14 min (40% A), 14–16 min (50% A) and 16–21 min (55% A) at a flow of 0.5 mL/min. The auto-sampler was conditioned at 15 °C, and the injection volume was 15 μL. 

The Q-TOF settings were as follows: nebulizer nitrogen gas with 220 °C temperature, 5.5 bar pressure, drying gas of 10 L/min, capillary voltage was set to 4.5 kV; end plate offset 500 V, transfer 200 Vpp, quadrupole ion energy 3 eV, collision cell energy of 7 eV e transfer time 72 µS. The micrOTOF_Q III was programmed for scanning low mass (100–380 *m*/*z*). 

The LC-QTOF/MS method was only validated for the quantification of LQFM05 (not for LQFM235) in plasma and tissues according to the Brazilian Health Surveillance Agency [22,29] guidelines: selectivity, linearity, precision and accuracy, recovery, matrix effect, carryover and stability of the analyte and IS were the analytical parameters assessed. For LQFM 235, the Q-TOF settings were kept as displayed on Table 5, although *m*/*z* transitions were set at 365 →173.

### 3.6. Analytical Validation

#### 3.6.1. Selectivity

Six different blank plasma batches and animal tissue homogenates (brain, heart, liver and kidneys) were investigated by assessing possible endogenous interferences at the same retention time of analyte and IS.

#### 3.6.2. Linearity

Linearity of the calibration range (10.0, 20.0, 25.0, 50.0, 100.0, 250.0, 400.0, 600.0, 750.0 and 900.0 ng/mL) was assessed by triplicate samples on two different days. Blank samples were spiked with LQFM05 and IS (analytical curve), and zero samples (blank samples spiked only with IS) of the biological matrix were also analyzed. 

Calibration curves were analyzed by the linear least squares regression method applied to a graphical plot of the analyte/IS peak intensity ratio (*y axis*) *versus* the theoretical concentration (*x axis*) of the analyte. Linearity was assessed by correlation (r) and determination (r^2^) coefficients and other additional criteria, such as, graphically, %RSDslope, studentized residual and homoscedasticity (Cochran’s hypothesis test and *F*-test values).

#### 3.6.3. Precision and Accuracy

Precision and accuracy were evaluated by spiked QC plasma samples in replicates (*n* = 6) at four concentration levels in the same day (intra-day repeatability). Intermediate precision and accuracy (inter-day) were also investigated at the same QC levels on two different days (*n* = 12). Precision was expressed as a percentage of the relative standard deviation (RSD%) of the specified concentrations, while accuracy was determined by the relative error (RE%) of the experimental samples [22].

#### 3.6.4. Recovery of the Extraction Procedure

Relative recovery was determined by comparison of analyte response in the spiked matrix samples before and after extraction procedure at all three QC levels (30.0, 450.0 and 750.0 ng/mL) [22,24].

#### 3.6.5. Carry-Over Effects

Carryover effect was assessed by comparing the chromatographic profile of blank samples’ homogenates spiked with LQFM05 (900.0 ng/mL). One blank plasma homogenate was run before spiked sample analysis and repeated twice after the upper concentration limit of quantification (900.0 ng/mL). 

#### 3.6.6. Matrix Effect

In order to evaluate the matrix effect, blank samples were spiked with IS (250 ng mL^−1^) and drug aliquots at three concentration levels (30.0, 450.0, 750 ng mL^−1^). Next, the drug/IS peak ratio was compared to drug response in solution in order to calculate the normalized matrix factor (NMF) for each sample, according to Equation (2) [29].

(2) $$NMF=\frac{LQFM05\;\mathrm{response}\;\mathrm{in}\;\mathrm{matrix}/\mathrm{IS}\;\mathrm{response}\;\mathrm{in}\;\mathrm{matrix}}{LQFM05\;\mathrm{response}\;\mathrm{in}\;\mathrm{solution}/\mathrm{IS}\;\mathrm{response}\;\mathrm{in}\;\mathrm{solution}}$$

#### 3.6.7. Stability

The stability of the analyte and IS was determined by the following tests: three freeze-thaw cycles, short-term stability and post-processing assay at two concentration levels (low and high QCs). Short-term stability samples were kept in an auto-sampler (15 °C) and analyzed after 35 h of storage. Post-processing stability samples were injected after being kept in the refrigerator (2–8 °C, 58 h), followed by the auto-sampler storage (15 °C, 24 h).

## 4. Conclusions

In the presented study, an LC-QTOF/MS method for pharmacokinetic and tissue biodistribution of a new prototype drug named LQFM05 was developed and duly validated. Different sample preparation techniques (SPE, LLE and PPE) were evaluated. The PPE method was preferred due to its simplicity, reduced cost, lower preparation time, higher sensitivity and accuracy. The LC-QTOF/MS method was shown to be linear over the therapeutic range, selective and sensitive for LQMF05 tissue biodistribution studies after *i.v.* administration in rats. 

The LQMF05 biodistribution study demonstrated fast and wide body distribution to all tissues analyzed. The greater exposure and tissue affinity were seen for kidneys, thus suggesting a potential extrahepatic clearance. Additionally, LQFM05 crosses the blood-brain barrier, reaching the target tissue with a high tissue-blood Kp (1.9) and half-life t_1/2β_ of 2.5 h, accounting for a proper pharmacodynamic effect. In addition, brain tissue showed the highest tissue concentration (Cmax: 12,357.0 ng/g), thus evidencing its higher tissue affinity rate.

In conclusion, the developed and validated LQFM05 method was successfully applied to different tissue samples. Accordingly, our results showed that LQFM05 is a promising antipsychotic drug, mainly metabolized in the liver.

## Figures and Tables

**Table 1:** Calibration curve data for LQFM05 (range of 10.0 to 900.0 ng/mL) at all studied matrices.

Parameter	Plasma	Brain	Heart	Liver	Kidneys
**Slope** ^a^ (±SD, *n* = 4)	1.761 × 10^−3^± 1.587 × 10^−5^	9.882 × 10^−4^ ± 8.168 × 10^−6^	1.239 × 10^−3^ ± 1.985 × 10^−5^	1.692 × 10^−3^ ± 7.928 × 10^−6^	1.127 × 10^−3^ ± 1.655 × 10^−5^
**Intercept** ^a^ (±SD, *n* = 4)	1.081 × 10^−3^ ± 7.043 × 10^−3^	0.905 × 10^−2^ ± 0.362 × 10^−2^	−0.7043 × 10^−3^ ± 0.8809 × 10^−2^	7.30 × 10^−3^ ± 3.518 × 10^−3^	8.292 × 10^−3^ ± 7.345 × 10^−3^
**r** ^2 b^	0.9969	0.9974	0.9903	0.9992	0.9919
**RSD**_slope_ (%) ^c^	0.901	0.827	1.602	0.469	1.469
**F**_calculated_ ^d^	1.351	1.257	0.285	1.062	0.641
C_calculated_^e^	0.201	0.226	0.204	0.073	0.191

^a^ Peak intensity of the analyte/IS vs. concentration. ^b^ Correlation coefficient. ^c^ Relative standard deviation of the slope (RSD_slope_ ≤ 2%). ^d^ Fisher ratio; F_cal_ < F_tab_ (linear relationship) = calculated and tabulated value (α = 0.05), respectively [18]. ^e^ Cochran test; C_cal_ < C_tab_ (homoscedastic residuals) = calculated and tabulated value (α = 0.05), respectively [19].

**Table 2:** Precision and accuracy of LQFM05 in rat plasma and tissue homogenates (brain, heart, liver and kidneys) by LC-QTOF/MS.

Matrix	C_spiked_ (ng/mL)	Intra (*n* = 6)			Inter (*n* = 12)		
		C_M_ Mean ± SD (ng/mL)	RSD%	RE%	C_M_ Mean ± SD (ng/mL)	RSD%	RE%
Plasma	10	9.66 ± 0.51	5.3	−3.4	9.98 ± 0.72	7.2	−0.2
	30	31.00 ± 1.37	4.4	3.3	30.71 ± 1.57	5.1	2.4
	450	455.58 ± 9.50	2.1	1.2	453.78 ± 8.97	2.0	0.8
	750	754.80 ± 10.30	1.4	0.6	754.24 ± 10.06	1.3	0.6
Brain	10	9.44 ± 0.56	6.0	−0.2	9.76 ± 0.79	8.1	−2.4
	30	29.31 ± 1.47	5.0	−0.1	29.36 ± 1.39	4.7	−2.1
	450	432.76 ± 3.20	0.7	−0.2	441.38 ± 9.34	2.1	−1.9
	750	727.68 ± 7.53	1.0	−4.2	743.67 ± 18.95	2.5	−0.8
Heart	10	9.85 ± 0.30	3.4	−1.5	9.92 ± 0.25	2.6	−0.8
	30	30.06 ± 0.12	0.4	0.2	30.22 ± 0.42	1.4	0.7
	450	451.04 ± 8.07	1.8	0.2	449.65 ± 6.04	1.3	−0.1
	750	750.11 ± 11.61	1.5	0.0	751.43 ± 9.65	1.3	0.2
Liver	10	10.22 ± 0.37	3.6	2.2	10.30 ± 0.35	3.4	3.0
	30	30.16 ± 0.86	2.9	0.5	30.36 ± 0.76	2.5	1.2
	450	449.78 ± 7.87	1.8	0.1	449.19 ± 6.16	1.4	−0.2
	750	751.24 ± 8.68	1.2	0.2	749.57 ± 8.92	1.2	−0.1
Kidneys	10	10.59 ± 0.71	6.7	5.9	10.49 ± 0.71	6.7	4.9
	30	29.84 ± 0.77	2.6	−0.5	29.94 ± 0.75	2.5	−0.2
	450	452.49 ± 10.95	2.4	0.6	453.60 ± 8.57	1.9	0.8
	750	756.41 ± 10.11	1.3	0.9	752.52 ± 8.93	1.2	0.3

C_spiked_: spiked concentration; CM: measured concentration; mean determination; SD: standard de-viation; RSD%: relative standard deviation percentage; RE%: relative error.

**Table 3:** Recovery and matrix effect data expressed by standard deviation (SD) and precision for all evaluated matrices (*n* = 6 for QC and *n* = 18 for IS) by LC-QTOF/MS.

Matrix		C_spiked_ (ng/mL)	Recovery		Matrix Effect (NMF)	
			Mean ± SD (%)	RSD (%)	Mean ± SD	RSD (%)
Plasma	LQFM05	30	55.53 ± 4.63	8.3	1.02 ± 0.09	8.8
		450	56.82 ± 5.0	8.7	1.04 ± 0.06	5.8
		750	56.22 ± 3.70	6.6	1.03 ± 0.03	2.9
	IS	250	79.50 ± 4.93	6.2	1.09 ± 0.04	4.1
Brain	LQFM05	30	45.84 ± 3.82	8.3	1.09 ± 0.04	4.1
		450	47.54 ± 2.03	4.3	1.05 ± 0.04	3.6
		750	43.26 ± 1.62	3.7	1.06 ± 0.03	2.7
	IS	250	94.38 ± 4.50	4.8	0.99 ± 0.07	7.3
Heart	LQFM05	30	68.94 ± 3.87	5.6	1.04 ± 0.04	3.5
		450	63.47 ± 1.84	2.9	1.00 ± 0.05	4.5
		750	65.28 ± 3.05	4.7	0.98 ± 0.04	4.4
	IS	250	99.61 ± 5.89	5.9	1.02 ± 0.05	5.1
Liver	LQFM05	30	71.82 ± 4.79	6.7	0.99 ± 0.04	3.7
		450	59.13 ± 2.92	4.9	0.98 ± 0.06	6.3
		750	70.75 ± 5.03	7.1	1.00 ± 0.04	3.7
	IS	250	82.38 ± 7.04	8.5	1.00 ± 0.03	3.0
Kidneys	LQFM05	30	63.77 ± 6.97	10.9	1.05 ± 0.04	3.6
		450	55.40 ± 2.53	4.6	1.06 ± 0.03	2.7
		750	66.50 ± 2.38	3.6	0.99 ± 0.07	7.3
	IS	250	98.31 ± 3.70	3.8	1.04 ± 0.04	3.5

IS: Diazepam; C_spiked_: spiked concentration; NMF: normalized matrix factor; mean determination; SD: standard deviation; RSD%: relative standard deviation percentage; QC: quality control samples.

**Table 4:** Tissue pharmacokinetic parameters after *i.v.* administration of LQFM05 (10 mg/Kg) to male Wistar rats (mean, *n* = 3) out of 21 rats randomly distributed into seven groups. Data obtained from Phoenix WinNonlin^®^ 8.1.

Matrix	AUC_(0–∞)_ (h*ng/g)	t_max_ (h)	C_max_ (ng/g)	K_β_ (h^−1^)	t_1/2_β (h)	MRT (h)	Kp ^b^
Plasma	5366.7 ^a^	-	-	0.31	2.3	1.7	-
Brain	10,460.6	0.08	12,357.0	0.28	2.5	2.0	1.9
Heart	10,919.6	0.08	7430.9	0.33	2.1	2.1	2.0
Liver	8235.3	0.17	4922.9	0.19	3.6	3.1	1.5
Kidneys	14,595.2	0.08	8768.0	0.30	2.3	2.4	2.7

^a^: h*ng/mL. ^b^ Kp: tissue/plasma ratio = AUC_(0_**_–_**_∞) tissue_/AUC_(0_**_–_**_∞) plasma_. AUC_(0-∞)_: area under the matrix concentration-time curve from time zero to infinity; t_max_: time to peak concentration; C_max_: peak concentration; K_β_: elimination rate constant; t_1/2_β: terminal half-life; MRT: mean residence time.

**Table 5:** MRM transition in positive ion mode, collision energy and retention time for the determination of LQFM05 and diazepam (IS).

No.	Compounds	MRM Transition (*m*/*z*)		CE *(eV)	RT ** (min)
		Quantifier	Qualifier		
1	Diazepam (IS)	285.08 → 257.09		20	5.6
2	LQFM05	349.21 → 157.08	349.21 → 191	15	6.3

* Collision energy; ** Retention time.

## Data Availability

Not applicable.
